# The leaf senescence-promoting transcription factor AtNAP activates its direct target gene *CYTOKININ OXIDASE 3* to facilitate senescence processes by degrading cytokinins

**DOI:** 10.1186/s43897-021-00017-6

**Published:** 2021-10-13

**Authors:** Youzhen Hu, Bin Liu, Huazhong Ren, Liping Chen, Christopher B. Watkins, Su-Sheng Gan

**Affiliations:** 1 Plant Biology Section, School of Integrative Plant Science, Cornell University, Ithaca, New York, 14853 USA; 2 Current address: College of Food Science, Shihezi University, Xinjiang, 832000 China; 3 College of Horticulture, China Agriculture University, Beijing, 100193 China; 4 Current address: Department of Plant Genomics, Centre for Research in Agricultural Genomics (CRAG), CSIC-IRTA-UAB-UB, Bellaterra, Spain; 5 College of Agriculture and Biotechnology, Zhejiang University, Hangzhou, 310058 China; 6 Horticulture Section, School of Integrative Plant Science, Cornell University, Ithaca, New York, 14853 USA

**Keywords:** ABA, ABA-cytokinin crosstalk, Aging, Arabidopsis, Cytokinin metabolism, Leaf longevity, Plant hormone, Senescence-associated genes (*SAG*s)

## Abstract

Cytokinins (CKs) are a class of adenine-derived plant hormones that plays pervasive roles in plant growth and development including cell division, morphogenesis, lateral bud outgrowth, leaf expansion and senescence. CKs as a “fountain of youth” prolongs leaf longevity by inhibiting leaf senescence, and therefore must be catabolized for senescence to occur. AtNAP, a senescence-specific transcription factor has a key role in promoting leaf senescence. The role of *AtNAP* in regulating CK catabolism is unknown. Here we report the identification and characterization of AtNAP-*AtCKX3* (*cytokinin oxidase 3*) module by which CKs are catabolized during leaf senescence in Arabidopsis. Like *AtNAP*, *AtCKX3* is highly upregulated during leaf senescence. When *AtNAP* is chemically induced *AtCKX3* is co-induced; and when *AtNAP* is knocked out, the expression of *AtCKX3* is abolished. AtNAP physically binds to the *cis* element of the *AtCKX3* promoter to direct its expression as revealed by yeast one-hybrid assays and *in planta* experiments. Leaves of the *atckx3* knockout lines have higher CK concentrations and a delayed senescence phenotype compared with those of WT. In contrast, leaves with inducible expression of *AtCKX3* have lower CK concentrations and exhibit a precocious senescence phenotype compared with WT. This research reveals that AtNAP transcription factor˗*AtCKX3* module regulates leaf senescence by connecting two antagonist plant hormones abscisic acid and CKs.

**Supplementary Information:**

The online version contains supplementary material available at 10.1186/s43897-021-00017-6.

## Core

Cytokinins retard leaf senescence and must be degraded at the onset of and during leaf senescence. A mechanism is revealed, in which the senescence-specific transcription factor, AtNAP, physically binds to the promoter of AtCKX3 to activate cytokinin oxidase expression and catabolizing cytokinins and thereby facilitating senescence processes.

## Introduction

Cytokinins (CKs) have pervasive roles in regulating many aspects of plant growth and development, and is involved in the response to stresses in plants (Gan and Amasino, 1996; Kieber and Schaller, 2014). The role of CK in inhibiting plant senescence has been investigated for decades (Gan 2010). Exogenous CK and senescence-specific production of endogenous CK biosynthesis led to the retardation of leaf senescence (Gan and Amasino, 1995; Gan and Amasino 1996). It was reported that CK-mediated delay in senescence was caused by increased sink strength via direct activation of extracellular invertase activity (Balibrea Lara et al., 2004; Zwack and Rashotte, 2013). CK concentrations decrease with progression of senescence in many plant species (Gan and Amasino, 1996; Noodén, 2012). It is known that during leaf senescence the expression of genes involved in CK biosynthesis in Arabidopsis decreases (Breeze et al., 2011) and the transcript levels of genes of CK-degrading enzymes increase (Guo et al., 2004; Breeze et al., 2011). The involvement of CKXs in leaf senescence and their regulatory mechanisms remain to be deciphered.

CKs, including isopentenyl adenine (IPA), zeatin (Z) and their ribosides, are a class of adenine-derived plant hormones that contain an isoprenoid or aromatic side chain in the sixth position of the purine ring. CKs are believed to be synthesized mainly via the key enzyme isopentenyl transferases (IPTs) (Gan and Amasino, 1996) and degraded by cytokinin oxidases (CKXs) (Armstrong, 1994; Avalbaev et al., 2012; Zhang et al., 2021). CKXs are flavin adenine dinucleotide–containing oxidoreductases that selectively cleave unsaturated N^6^ side chains from IPA or Z (Armstrong, 1994; Jones and Schreiber, 1997), and are responsible for the irreversible degradation and inactivation of CK in many plant species (Mok and Mok, 2001; Avalbaev et al., 2012; Zhang et al., 2021). Both IPTs and CKXs play pivotal roles in the CK homeostasis that is critical for plant growth and development.

Leaf senescence is a genetically and epigenetically programmed process that can be induced by internal and adverse environmental factors, that is driven by the expression of *senescence-associated genes* (*SAG*s), and involves nutrient recycling (Gan and Amasino, 1997; Yuan et al., 2020; Guo et al., 2021). ~ 10% of genes in the Arabidopsis genome is expressed during leaf senescence, including > 130 transcription factor (TF) genes and > 180 genes encoding proteins/enzymes involved in signal transductions (Guo et al., 2004; Breeze et al., 2011). TFs are proteins that often bind to special DNA sequences (named motifs) of genes’ promoters to activate or repress the expression of the genes at the transcription level. Various TFs such as NAC, WRKY, C2H2, APE2, MYB, HB, and bZIP have been shown to play critical roles in leaf senescence (Guo et al., 2004; Guo et al., 2021). For example, the ABA-regulated AtNAP (a member of NAC superfamily TF) and its homologs regulate leaf senescence in Arabidopsis (Guo and Gan, 2006), Chinese cabbage (Li et al., 2021), rice (Liang et al., 2014), wheat (Uauy et al., 2006), and cotton (Fan et al., 2015), and AtNAP binds to a 9 bp *cis* element to active the expression of *SAG113*, a gene encoding a protein phosphatase 2C, to promote leaf senescence (Zhang and Gan, 2012; Zhang et al., 2012). Whether and how the NAP TF regulate CKXs and thus cytokinin levels are unknown.

Here we report that *SAG202* encoding AtCKX3 is responsible for the degradation of CKs at the onset of and during leaf senescence in Arabidopsis, and that AtNAP binds to the promoter of *AtCKX3* to activate the senescence-specific expression of this CK-degrading gene.

## Results

### *AtCKX3* expression is leaf senescence specific

**Fig. 1:**
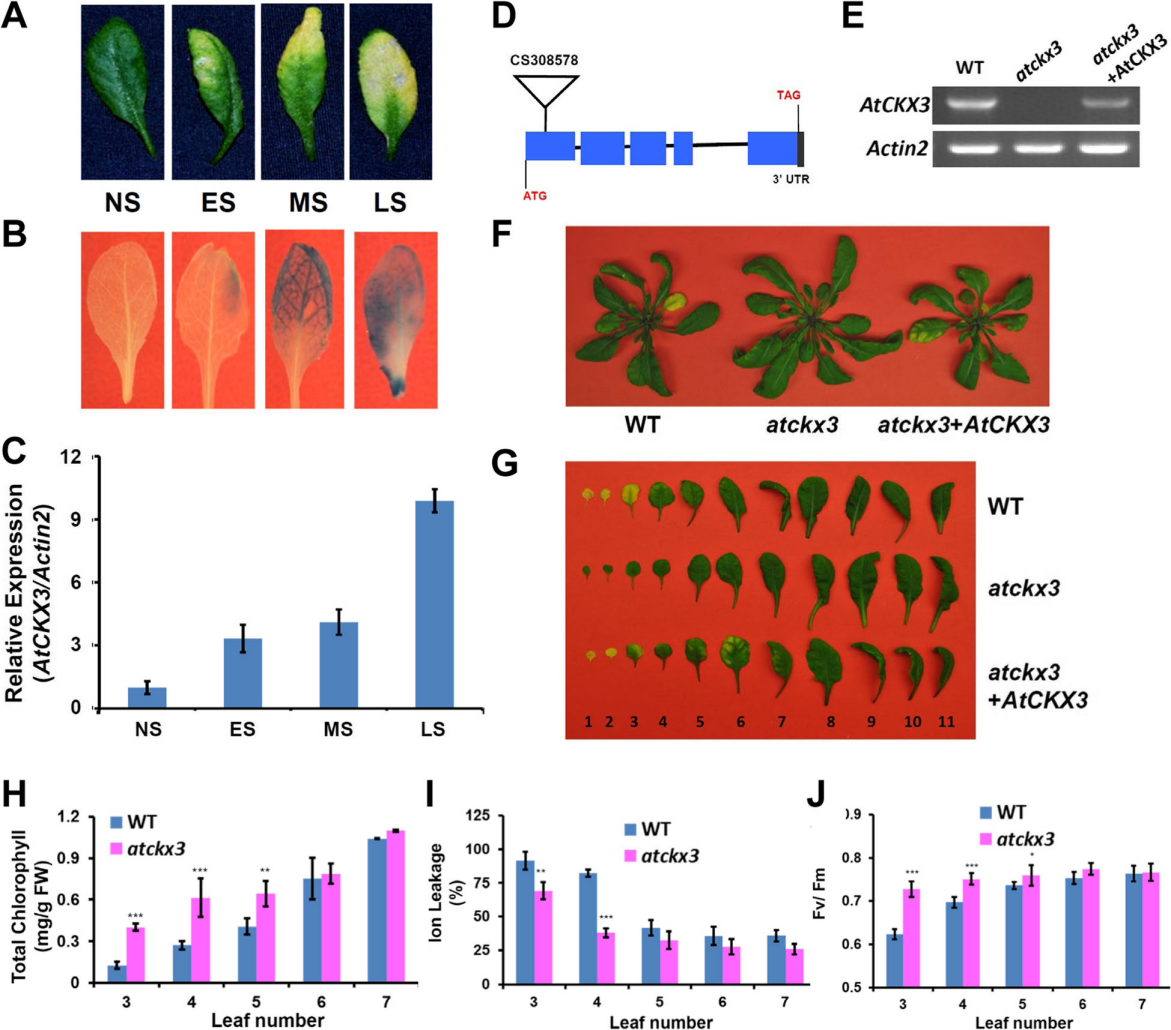
**Phenotypical, physiological and molecular genetic analyses of** ***AtCKX3*** **(a.k.a.** ***SAG202*).** (A) Leaves at different stages of senescence. NS, non-senescence; ES, early senescence with < 25% leaf yellowing; MS, mid-senescence with ~ 50% leaf yellowing; LS, late senescence with > 75% leaf yellowing. (B) GUS staining of the P_*AtCKX3*_-*GUS* transgenic leaves at different senesce stages shown in (A). (C) qPCR analysis of the *AtCKX3* expression during senescence in wild type (WT) leaves. Data indicate average values ± SD of three samples. (D) Gene structure of *AtCKX3* and T-DNA insertion site in the first exon region of gene in Arabidopsis line CS414980 (designated as *atckx3*). (E) RT-PCR analyses of *AtCKX3* transcripts in senescent leaves of WT, *atckx3* (CS414980) and a complementation line (*atckx3* + *AtCKX3*). (F) Age-matched rosette leaves of WT, *atckx3* and the complementation line (*atckx3* + *AtCKX3*). (G) Leaves detached from the age-matched plants in (F). Leaves were counted from bottom with the oldest leaf as 1 and the youngest leaf 11. Pictures were taken at 35 DAG (days after germination). Measurements of chlorophyll contents (H), ion leakage (I), and *F*_v_/*F*_m_ ratios in age-matched leaves (no. 3 through no. 7) of WT and atckx3 plants (35 DAG). FW, fresh weight. Error bars represent SD of six repeats. *, ** and *** represent significant differences of *P* < 0.05, *P* < 0.01 and *P* < 0.001, respectively. Student’s t-test was used

We previously established a leaf senescence transcriptome in Arabidopsis (Guo et al. 2004), among which is *SAG202* (AT5G56970) that encodes cytokinin oxidase 3 (AtCKX3). Quantitative PCR (qPCR) analyses revealed that the transcript levels of *AtCKX3* were very low in non-senescing leaves such as the expanding young leaves and the fully expanded mature leaves, and increased with the progression of senescence (Fig. 1 A and C). The *AtCKX3* promoter (P_*AtCKX3*_) was used to direct the expression of the reporter gene *GUS*. The GUS staining of the P_*AtCKX3*_-*GUS* transgenic plant (Fig. 1 B) further confirmed the leaf senescence-specific expression of *AtCKX3*.

### Leaf senescence is delayed in the *atckx3* knockout

To investigate the biological function of *AtCKX3* in leaf senescence, we obtained an Arabidopsis line named CS308578 from the Arabidopsis Biological Resource Center; this line contained a T-DNA inserted in the first exon of *AtCKX3* that abolished its expression in the homozygous mutant (Fig. 1D and E). The *atckx3* knockout plants exhibited a delayed leaf senescence phenotype (Fig. 1F), and there were no obvious differences in growth and development prior to senescence between the null mutants and wild type (WT) (Fig. 1G). Further analyses of physiological changes in total chlorophyll concentrations (Fig. 1H), ion leakage (Fig. 1I) and ratio of variable to maximum chlorophyll fluorescence from photosystem II (*F*_v_/*F*_m_) (Fig. 1J) also showed delayed leaf senescence of the null plants. The *F*_v_/*F*_m_ ratio represents the maximum potential quantum efficiency of Photosystem II if all capable reaction centers are open; a leaf is likely undergoing senescence if the ratio is less than 0.7 (Gan 2010).

To ensure the delay in leaf senescence described above was due to the knockout of *AtCKX3*, we performed a complement experiment by introducing a copy of *AtCKX3* from WT into the *atckx3* null mutant plants. The expression of *AtCKX3* in the complement plants was restored to WT (Fig. 1E) and leaves senesced as those of WT (Fig. 1F and G).

### Inducible overexpression of *AtCKX3* drastically accelerates leaf senescence

**Fig. 2:**
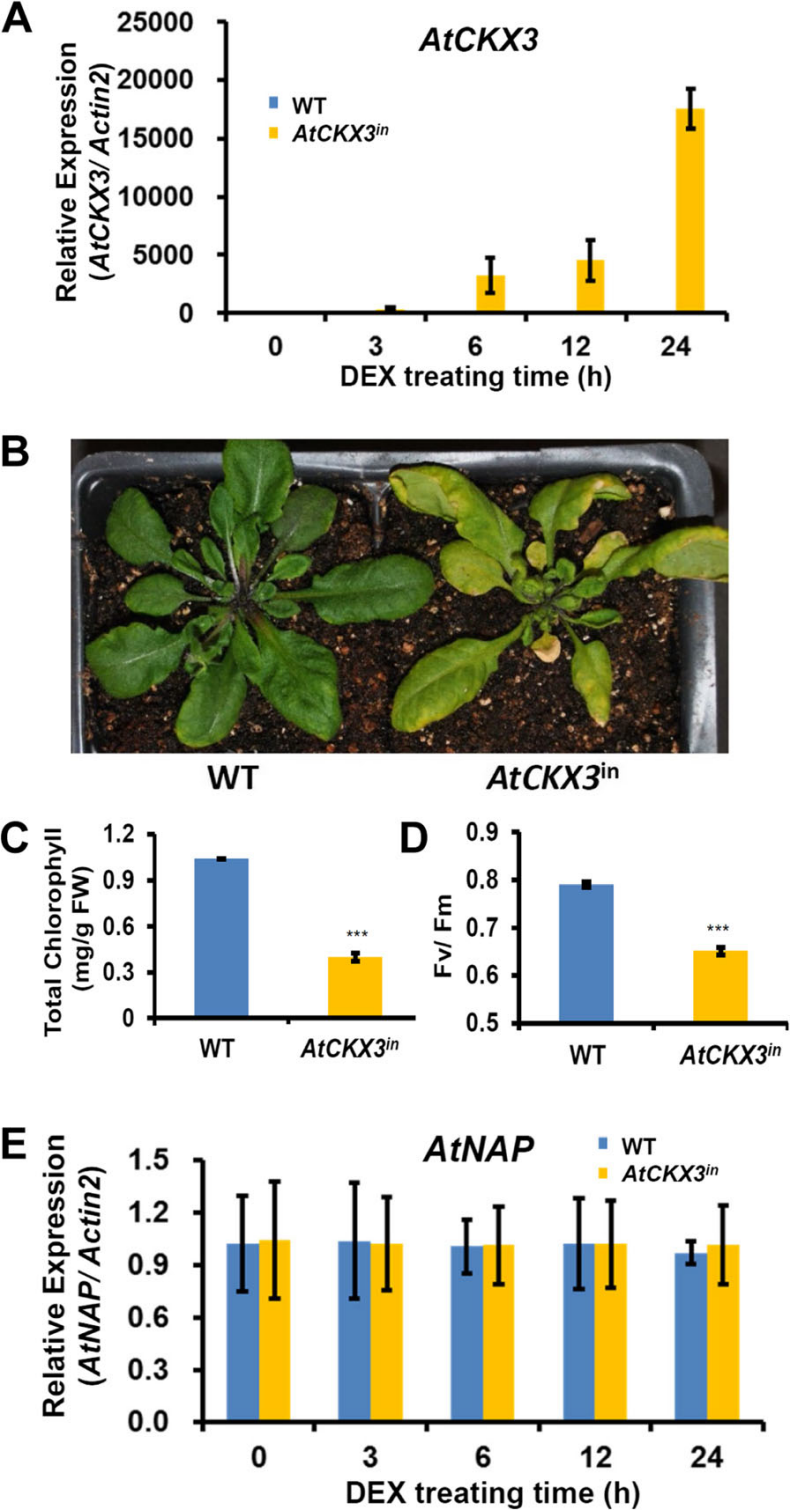
**Precocious leaf senescence caused by dexamethasone (DEX)-induced** ***AtCKX3*** **overexpression**. (A) Transcript levels of *AtCKX3* in WT and *AtCKX3*^in^ transgenic plants at different time points after DEX induction were qPCR analyzed. Data indicate mean values ± SD of three samples. (B) Phenotypes of WT and DEX-induced *AtCKX3* transgenic plant. Photo was taken 5 days after DEX treatment of plants (21 DAG). Total chlorophyll contents (D) and *F*_v_/*F*_m_ ratios (E) of leaves from WT and *AtCKX3*^in^ plants 24 h after DEX treatment were measured. Data indicate mean values ± SD of six samples. *** represent significant differences of *P* < 0.001. Student’s t-test was used. (E) Transcript levels of *AtNAP* in WT and *AtCKX3*^in^ transgenic plant at different time points after DEX induction were qPCR analyzed. Data indicate mean values ± SD of three samples

*AtCKX3*’s role in leaf senescence was further investigated in gain-of-function mutants. Considering that CKs have pivotal roles in early plant growth and development, and that constitutive overexpression of a cytokinin oxidase gene may result in abnormal or dead plants, we used the dexamethasone (DEX, a synthetic glucocorticoid) inducible overexpression system (Aoyama and Chua 1997). As shown in Fig. 2A, the transcript levels of *AtCKX3* in leaves of the *AtCKX3*^in^ transgenic plants increased with time after DEX treatment, resulting in a visible precocious leaf senescence phenotype (Fig. 2B) that was consistent with the reduced contents of chlorophyll levels (Fig. 2C) and decreased *F*_v_/*F*_m_ ratio (Fig. 2D) compared with those of WT. We also analyzed the expression levels of *AtNAP* in the *AtCKX3*^in^ transgenic lines, and found that the induced *AtCKX3* expression did not alter the *AtNAP* expression (Fig. 2E).

### *AtCKX3* modulates the cytokinin contents during leaf senescence

**Fig. 3:**
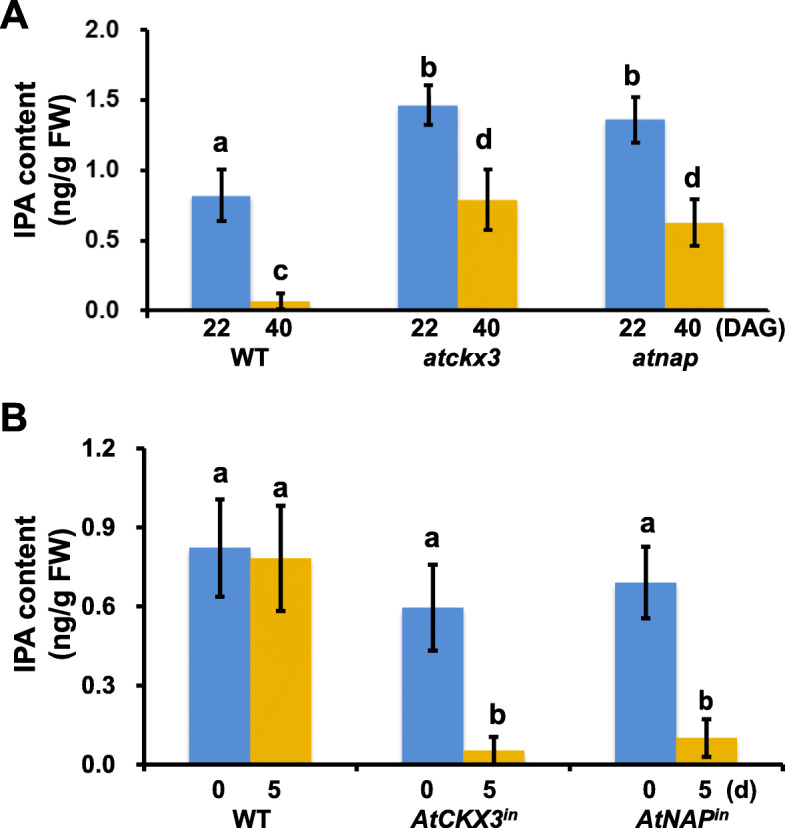
**LC-MS/MS analyses of isopentenyladenine (IPA) levels in WT,** ***atckx3*,** ***atnap*,** ***AtCKX3***^**in**^ **and** ***AtNAP***^**in**^**.** (A) IPA levels in the 5th leaves of WT, *atckx3* and *atnap* plants at 22 DAG) (leaves have not started senescing) and 40 DAG (the leaves are senescing in WT but not the mutants), respectively. (B) IPA levels in fully expanded leaves of WT, *AtCKX3*^in^ and *AtNAP*^in^ plants 0 and 5 days after DEX treatment. Significant (*P* < 0.05) differences between means are indicated by different letters. ANOVA analysis with LSD test was used

CKs have been shown to retard leaf senescence (Gan and Amasino 1995). We hypothesized that as a member of CK oxidases, AtCKX3 promoted leaf senescence by catalyzing the degradation of CKs. We therefore utilized LC-MS/MS (Pan et al., 2008) to determine the concentrations of isopentenyladenosine (IPA, a species of CKs) in young and senescing plants of WT, *atckx3* and *atnap* null mutants (Fig. 3A), and in leaves before and after DEX-induced *AtCKX3* and *AtNAP* overexpression lines (Fig. 3B). AtNAP is a leaf senescence-specific NAC family transcription factor (TF) (Guo and Gan, 2006) that was shown to directly regulate *AtCKX3* expression below. These data suggested that AtCKX3 could degrade IPA and thereby facilitate leaf senescence.

### *AtCKX3* is positively regulated by AtNAP TF

**Fig. 4:**
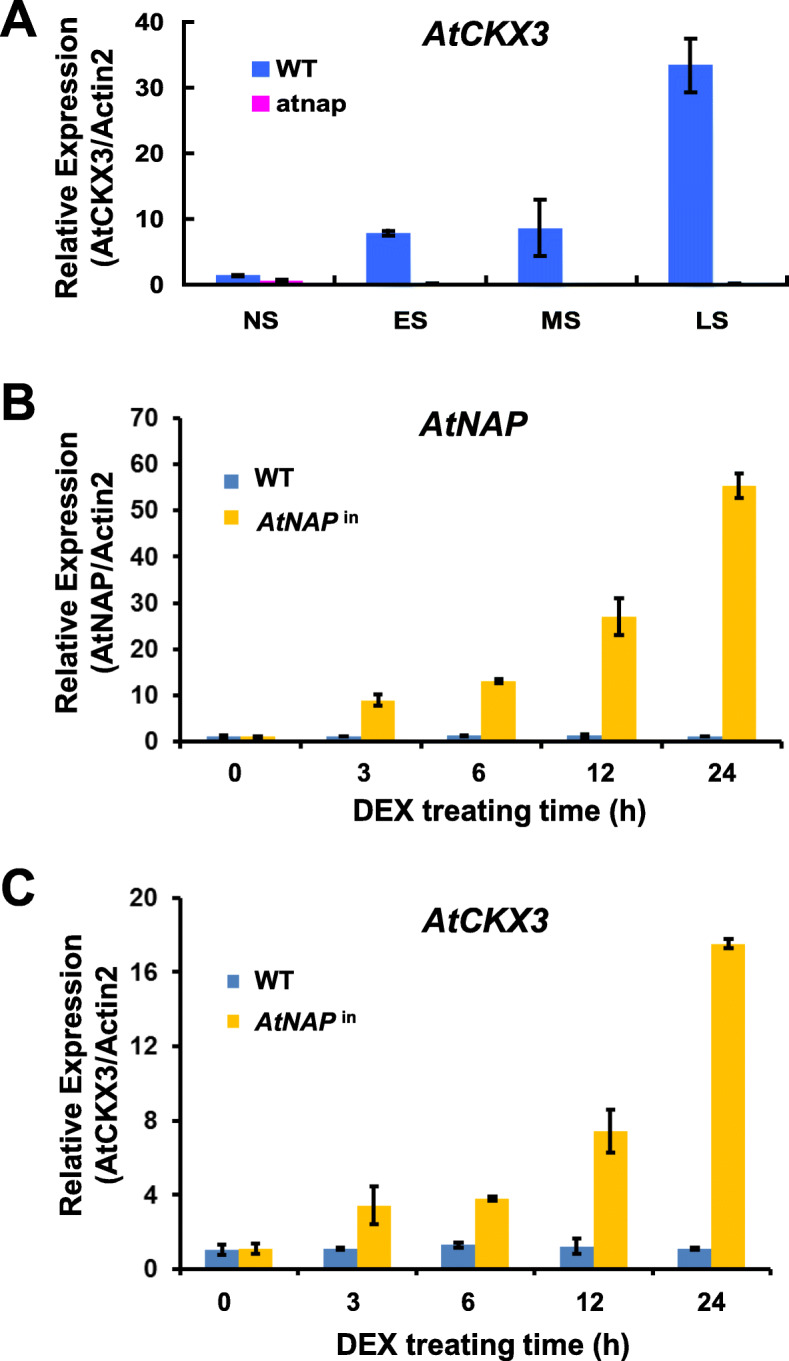
**qPCR analyses of** ***AtCKX3*** **in** ***atnap*** **and** ***AtNAP***^**in**^**.** (A) Diminished expression of *AtCKX3* in *atnap* plants. (B) Induction of *AtNAP* transcripts in leaves of *AtNAP*^in^ plants treated with DEX. (C) Co-induction of *AtCKX3* transcripts in the same leaves of *AtNAP*^in^ plants treated with DEX shown in (B). Data indicate mean values ± SD of three samples

We were interested in the regulatory mechanism underlying the *AtCKX3* expression, and we hypothesized that it might be regulated by AtNAP. First, we investigated the expression patterns of *AtCKX3* in the *atnap* null and *AtNAP*-inducible lines, respectively. The *AtCKX3* transcripts were barely detectable when *AtNAP* was knocked out (Fig. 4A). In contrast, when *AtNAP* was induced to express after DEX treatment (Fig. 4B), *AtCKX3* was co-induced (Fig. 4C).

### *AtCKX3* is a direct target of AtNAP TF in yeast and *in planta*

**Fig. 5:**
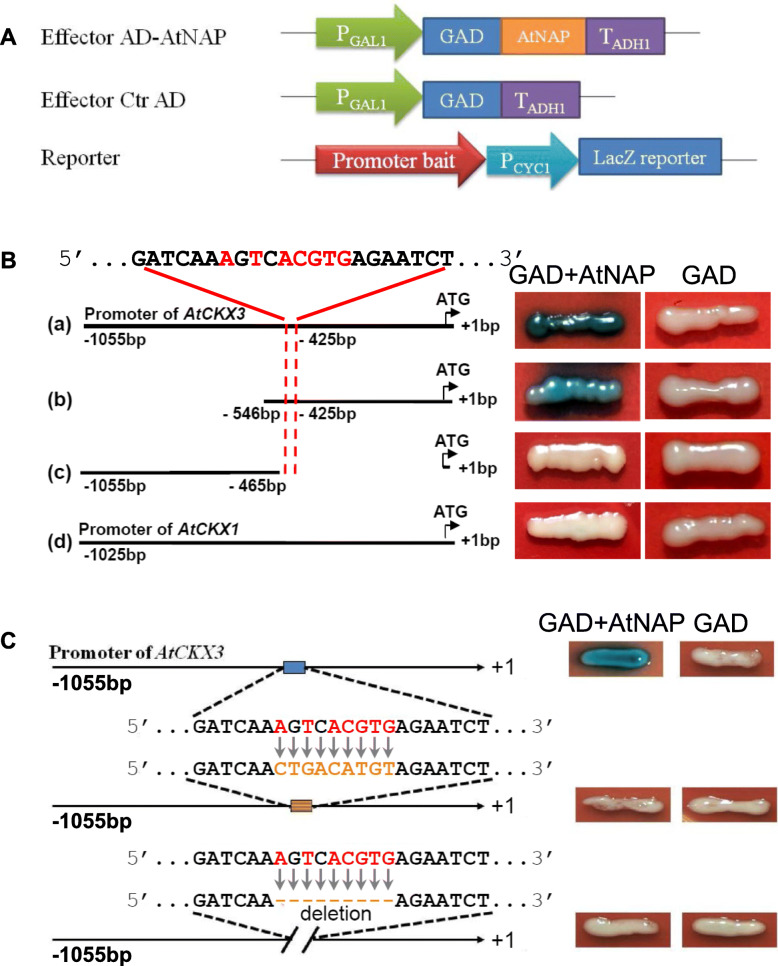
**Physical interaction of AtNAP with the** ***AtCKX3*** **promoter revealed by yeast one-hybrid assay.** (A) Schematic representation of the constructs in the studies. The expression of prey GAD fused with or without (negative control or Ctr) AtNAP were driven by the *GAL1* promoter (P_GAL1_). The LacZ gene driven by *AtCKX3* promoter or its truncations or mutations served as the bait. (B) Activation of the LacZ reporter by binding of the fusion protein GAD-AtNAP to the *AtCKX3* promoter. The *AtCKX1* promoter lacked the motif and served as the other control. Blue color suggests a binding. The sequence in red represents the motif which AtNAP binds to. A of the translation start site was numbered as + 1. (C) Failure of AtNAP binding to the *AtCKX3* promoter either containing transversion mutation or deletion of the motif sequence

We then investigated whether AtNAP TF directly regulated *AtCKX3* by binding to the gene promoter via both yeast one-hybrid analyses and *in planta* reporter gene expression approaches. The AtNAP TF bound to the 1055 bp and 546 bp promoter regions upstream of the *AtCKX3* start codon ATG but not the 591 bp DNA fragment (− 1055 bp ˗ -465 bp) (Fig. 5B). Sequence analysis revealed that the 464 bp promoter region of *AtCKX3* contained 5’AgTcACGTG3’ (− 433 bp ˗ -425 bp). The sequence was very similar to 5’AcTtACGTG3’, the complementary sequence of 5’CACGTAAGT3’ of the *SAG113* promoter that the AtNAP TF bound to (Zhang and Gan, 2012). We hypothesized that AtNAP TF binds to 5’AgTcACGTG3’ to activate *AtCKX3* expression. The hypothesis was confirmed by the fact that the *AtCKX1* promoter did not contain the sequence and showed no interaction (Fig. 5B). The hypothesis was further confirmed by changing the 9 bp sequence by transversion mutations or simply deleting the 9 bp sequence of the *AtCKX3* promoter and using the mutated promoters to perform yeast one-hybrid assays (Fig. 5C).

**Fig. 6:**
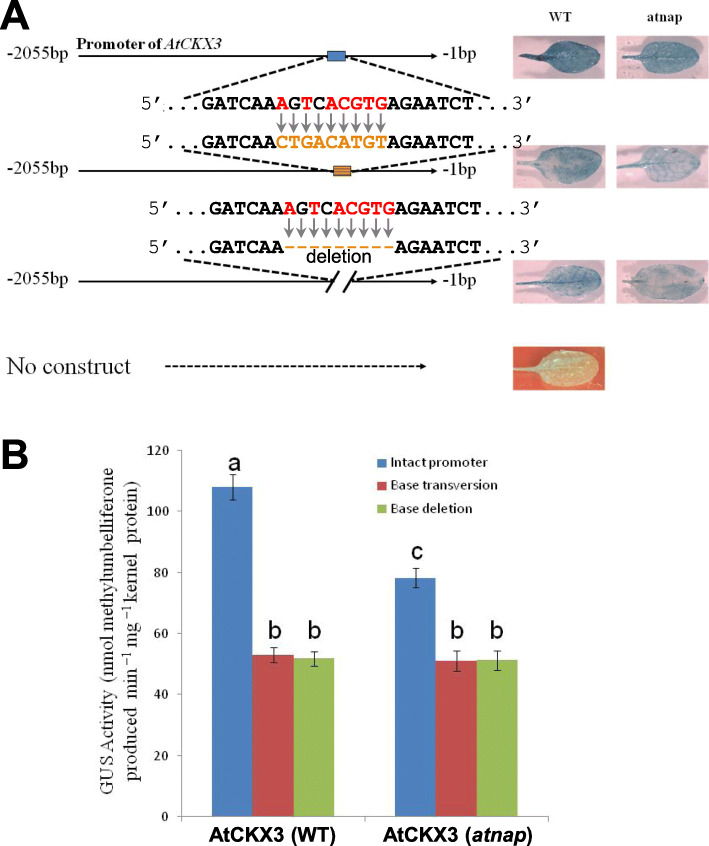
**Physical interaction of AtNAP with the** ***AtCKX3*** **promoter** ***in planta*.** (A) GUS staining of senescent leaves in transgenic Arabidopsis plants. The *AtCKX3* promoter and its mutant versions with transversion mutation or deletion of the 9-bp motif were used to direct the reporter *GUS* expression in WT and *atnap* null mutant, respectively. (B) GUS enzymatic activities of senescent leaves of various transgenic plants shown in (A). Data indicate mean values ± SD of five samples. Significant (*P* < 0.05) differences between means are indicated by different letters. ANOVA analysis with LSD test was used

We further investigated if AtNAP TF bound to the 9 bp sequence 5’AgTcACGTG3’ to activate the *AtCKX3* expression *in planta*. The *AtCKX3* promoter and its muted versions (with transversion mutations or deletion of the 9 bp sequences) were fused to the *GUS* reporter genes, and transferred into WT and *atnap* mutant plants, respectively. The *GUS* expression directed by the muted promoters or in the *atnap* background was reduced in terms of the GUS blue staining (Fig. 6A) and GUS enzyme activities (Fig. 6B), suggesting that AtNAP TF binds to 5’AgTcACGTG3’ to activate *AtCKX3* in senescing leaves *in planta*.

## Discussion

**Fig. 7:**
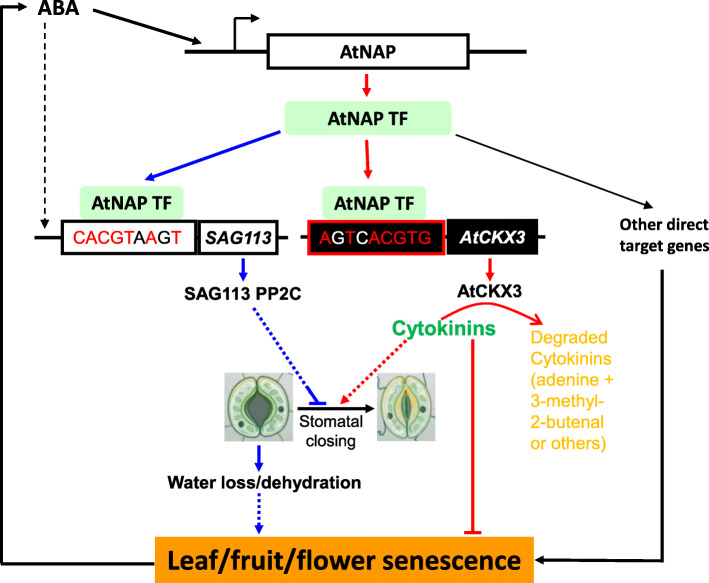
**ABA-*AtNAP*-*AtCKX3*-cytokinins in leaf senescence.** The senescence hormone ABA induces the expression of *AtNAP*, a NAC family transcription factor (TF) gene. AtNAP physically binds to the motif 5’AgTcACGTG3’ of the *AtCKX3* promoter to activate the gene expression to degrade cytokinins presented in the leaf; CK has been shown to inhibit leaf senescence (possibly via promoting stomatal closure), and the enzymatic removal of CK will facilitate the senescence processes. One the other hand, AtNAP has previously been shown to physically bind to the motif 5’CACGGTaAgT3’ of the *SAG113* promoter to direct the protein phosphatase 2C expression, which in turn prevents stomatal from closure, leading to water loss and senescence. It should be noted that the motif of one gene promoter is very similar to the complementary sequence of the other gene’s motif

It is well known that CKs inhibit leaf senescence (Gan and Amasino, 1995; Gan and Amasino, 1996; Gan and Amasino, 1997; Gan, 2010; Guo et al., 2021), and that the CK concentrations decrease rapidly at the onset of and during senescence in leaves and flowers (Gan and Amasino, 1996; Zou et al., 2021). Our research revealed a novel ABA-CK crosstalk mechanism by which the ABA-induced TF AtNAP physically binds to the promoter of *AtCKX3* to activate the expression of the enzyme that subsequently catabolizes CKs and facilitates the senescence processes (Fig. 7).

CKs regulate many processes including cell division, shoot initiation, chloroplast development, leaf expansion, organ size, and lateral bud growth (Kieber and Schaller, 2014; Ding et al., 2015; Di Marzo et al., 2020) and post-apical dominance suppression inhibition of lateral buds and inhibition of leaf senescence (Guo and Gan, 2011; Davies and Gan, 2012). However, CK present in leaves must be catabolized to facilitate leaf senescence. That *AtCKX3* is most likely responsible for the CK catabolism is supported by three lines of evidence: (1) *AtCKX3* is expressed at the onset of and during leaf senescence (Fig. 1); (2) the levels of IPA (a species of CKs) is higher in leaves of the *atckx3* knockouts than in age-matched WT plants (Fig. 3A); and (3) when *AtCKX3* is chemically induced in fully expanded but non-senescing leaves the IPA concentrations are reduced (Fig. 3B). The increase and decrease in IPA concentrations in the *atckx3* and *AtCKX*^in^ mutants result in delayed and precocious leaf senescence phenotypes, respectively (Fig. 2D and Fig. 3A), which also supports the important role of the enzymatic removal of CKs by AtCKX3 in facilitating senescence processes. *AtCKX3* was expressed in the central WUSCHEL domain to regulate the CK concentrations and thereby the activity of the reproductive meristems of Arabidopsis (Bartrina et al., 2011). *AtCKX3* was also expressed in the boundary between shoot apical meristem and the newly formed organ primordia to lower the CK concentration and to reduce cell division (Ding et al., 2015). Our research reveals the gene’s new functionality in leaf senescence.

The mechanism by which *AtCKX3* is regulated was also revealed by this research. We found that *AtCKX3* is a direct target gene of the AtNAP TF. Firstly, *AtCKX3* is co-expressed with *AtNAP*. When *AtNAP* is knocked out, the expression of *AtCKX3* is not detectable in leaves at various senescence stages (Fig. 1C and Fig. 4A); whereas when *AtNAP* is chemically induced (Fig. 4B), *AtCKX3* is also induced (Fig. 5C). Secondly, yeast one-hybrid experiments show that AtNAP physically binds to the motif 5’AgTcACGTG3’ at − 440 ~ − 432 bp (counted from the start codon ATG) of the promoter of *AtCKX3* (Fig. 5). Thirdly, AtNAP is also shown to physically interact with the very same motif 5’AgTcACGTG3’ of the *AtCKX3* promoter *in planta* (Fig. 6). When the motif sequence is replaced with transversion mutant sequence or is deleted, AtNAP can no longer bind to it to produce blue color in yeasts (Fig. 5C) or the binding is reduced *in planta* (Fig. 6). It has been shown that AtNAP binds to a 9-bp core sequence of the *SAG113* promoter, 5’CACGTAAGT3’ to direct the expression of the protein phosphatase 2C to promote leaf senescence in Arabidopsis (Zhang and Gan, 2012; Zhang et al., 2012). The complementary sequence of the motif on the *AtCKX3* promoter, 5’CACGTgAcT3’, is similar to the core binding sequence of the *SAG113* promoter, suggesting that the 7 bp (in capital) of the 9-bp core sequence is essential for the AtNAP TF to interact with. It is interesting to note that the *AtNAP*-*AtCKX3* module revealed here is quite distinctive from the *HANABA TARANU (HAN)*-*AtCKX3* shown in a previous study (Ding et al., 2015). In that study, a chromatin immunoprecipitation (ChIP)-PCR assay indicated that HAN binds to 5′WGATAR3’ (W: A or T; R: A or G) located at − 1619 ~ − 1614 bp of the *AtCKX3* promoter and at 1569 ~ 1574 bp of the gene’s first intron to activate the *AtCKX3* transcription (Ding et al., 2015).

The AtNAP-*AtCKX3* regulatory module connects two antagonist plant hormones ABA and CKs. ABA is known to promote senescence in leaves, fruits and flowers (Kou et al., 2012; Zhang and Gan, 2012; Zhang et al., 2012; Liu et al., 2016; Zhao et al., 2016; Guo et al., 2021) by inducing NAP TF in various plant species such as Arabidopsis (Zhang and Gan, 2012; Zhang et al., 2012), rice (Liang et al., 2014), and Chinese flowering cabbage (Li et al., 2021). The senescence-specific AtNAP TF have a pivotal role in positively regulating leaf and fruit senescence (Guo and Gan, 2006; Kou et al., 2012) via activating its direct target gene *SAG113* that encodes a protein phosphatase (PP) 2C (Zhang and Gan, 2012; Zhang et al., 2012). The PP2C then keeps stomata open to transpire, and the increased water loss leads to senescence (Zhang and Gan, 2012). We now reveal that AtNAP TF can also facilitate the senescence processes by activating its direct target *AtCKX3* to degrade CKs. Similar to the AtNAP-*AtCKX3* module, the RhNAP-*RhCKX3* module in rose has been reported to regulate petal senescence by degrading CKs; *RhNAP* in rose is an orthologue of *AtNAP* (Zou et al., 2021). We also found that Both OsNAP in rice and AtNAP could bind to the promoter of *OsCKX8* (but not *OsCKX9*) in rice as revealed by the yeast one-hybrid analyses (Supplemental Fig. S2). Whether the ABA-NAP TF-CKX-CK mechanism is ubiquitous for senescence in leaves, fruits and flowers are yet to be investigated.

It should be noted that there are 6 *CKX*s (*AtCKX1*–*6*) in Arabidopsis genome, and only *AtCKX1* and *AtCKX3* are up-regulated during leaf senescence (Supplemental Fig. S1). AtNAP failed to physically interact with the promoter of *AtCKX1* (Fig. 5B), and the senescence-associated upregulation of *AtCKX1* may involve a different mechanism from the *AtNAP*-*AtCKX3*-CKs module reported here.

Last, our research provided a new approach to prolonging pre- and post-harvest longevity of leaves, flowers and fruits by knocking out senescence-specific *CKX*s in horticultural and agronomic crops. A recent study showed that nullifying *OsCKX11* could significant delay leaf senescence and increase grain number in rice (Zhang et al., 2021), which supports the feasibility of the new approach in plant biotechnology.

## Materials and methods

### Plant materials and growth conditions

*A. thaliana* ecotype Columbia-0 (Col-0) was used as WT in the research. The *atnap* knockout mutants and the *AtNAP*-inducible expression lines were in Columbia background (Guo and Gan, 2006). The T-DNA insertion line CS308578 (designated as *atckx3*) was obtained from Arabidopsis Biological Resource Center (ABRC). Seeds were surface sterilized with three rinses in 70% ethanol containing 0.01% Triton X-100, and then sown on Petri dishes containing one-half-strength Murashige and Skoog salts with appropriate antibiotics when necessary. The plates were kept at 4 °C for 2 d before being moved to a growth chamber. Approximately 7 d after germination (DAG), Seedlings were transplanted to Cornell mix soils (3:2:1 peat moss:vermiculite:perlite, v/v/v). WT, mutants, and/or transgenic plants were grown side by side in the same tray at 22 °C with 60% relative humidity under constant light (120 μmol·m^− 2^·s^− 1^ light from a mixture of fluorescent and incandescent bulbs).

### Plasmid construction

To construct the inducible *AtCKX3* overexpression plasmid, the *AtCKX3* gene was amplified from Col-0 using primers G3945 and G3946, and subsequently cloned into an inducible binary vector pGL1152 (Guo and Gan, 2006). For complementation test, the DNA fragment including the promoter and coding sequence of AtCKX3 was cloned into the binary vector pPZP211 at the Xho I and Pst I sites (Hajdukiewicz et al., 1994). To make the P_*AtCKX3*_-*GUS* construct, the *AtCKX3* promoter (P_*AtCKX3*_) PCR-amplified with primers G3947 and G3948 was first cloned into pGEM-T Easy vector (Promega, USA), released with Pst I and Nco I, and subsequently cloned into intermediate vector pSG506 (Gan, 1995) to form the P_*AtCKX3*_-*GUS*. The recombinant gene was finally cloned into the binary vector pPZP211 (Hajdukiewicz et al., 1994). For complementation test, the *AtCKX3* CDS (3314 bp) was amplified with primers G4042 and G4043, the P_*AtCKX3*_ region (1267 bp) was amplified with primer G4044 and G4045, these two fragments were cloned into pGEM-T Easy vector (Promega, USA) separately, then released with Kpn I and Hind III, and the complementation construct including the promoter and coding sequence of *AtCKX3* was subsequently cloned into the binary vector pPZP211 (Hajdukiewicz et al., 1994). For the yeast (*Saccharomyces cerevisiae*) one-hybrid assay related constructs, the effectors with or without AtNAP were described previously (Guo and Gan, 2006; Zhang et al., 2012). served as a precursor constructed and preserved in our lab (Zhang and Gan 2012). To construct various *AtCKX3* promoter-*LacZ* reporters, the 1054-bp and various truncated or transversion or deletion mutant promoters of *AtCKX3* were PCR amplified and cloned into pLacZi2μ vector at the *Eco* RI and *Xho* I sites (Guo and Gan, 2006; Zhang and Gan, 2012). As a negative control, 1037 bp promoter region of *AtCKX1* was similarly constructed. To obtain transversion or deletion mutant promoters of AtCKX3, two pairs of primers (G4387 and G4388 with transversion mutation, G4389 and G4390 with deletion mutation) were designed, we used G4010 and G4387 to clone first fragment (436 bp) of AtCKX3 promoter, then the second part (634 bp) of AtCKX3 promoter was amplified by G4388 and G4011, after that, PCR products of the two fragments (they have 15 bp overlap region) were used as template to amplify the 1055 bp AtCKX3 promoter with transversion mutation. The same protocol was used to obtain AtCKX3 promoter with deletion mutation. All the primers used in this study were listed in Supplemental Table S1.

### Yeast one-hybrid assay

Yeast one-hybrid assays were performed as described by (Guo and Gan, 2006; Zhang and Gan, 2012). The plasmid with GAD-AtNAP fusion (pGL3175) was co-transformed with individual reporter constructs into the yeast strain EGY48. Transformants were grown on proper drop-out plates containing 5-bromo-4-chloro-3-indolyl-*β*-d-galactopyranoside for the blue color development.

### Histochemical GUS staining, chlorophyll contents, *F*_v_/*F*_m_ assay, and ion leakage measurement

Chlorophyll, fluorescence, and ion leakage analyses as well as histochemical GUS staining were performed as described previously (Guo and Gan, 2006; Zhang and Gan, 2012).

### Transcript analyses

RNA extraction, complementary DNA synthesis, and qPCR analysis were carried out as described previously (He and Gan, 2001; Gan, 2012). First-strand complementary DNA was synthesized from 3 μg of total RNA (treated with RNase-free DNase; New England Biolabs, USA) at 42 °C with MV-Reverse Transcriptase (Promega, USA). For qPCR, 1 μL of each diluted sample (40 folds) was used as a template in a 25-μL reaction. All qPCRs were performed on a Bio-Rad IQ-5 thermocycler with an annealing temperature of 55 °C. Cycle threshold values were determined by IQ-5 Bio-Rad software assuming 100% primer efficiency. Three mRNA samples from three independently harvested leaf samples were qPCR analyzed.

### Arabidopsis transformation

Various binary vectors were transferred into *A. tumefaciens* strain ABI. The transformed agrobacterial cells were used to transform Arabidopsis plants via floral dipping (Clough and Bent, 1998). Approximately 30 T1 transgenic lines for each transgene were selected, and the phenotypic and physiological analyses were performed in the T2 or subsequent generation homozygous for the transgene.

### CK quantification by coupled spectrophotometric assay

Leaf sample (0.1–0.2 g) of various plants were collected for analysis of IPA (a species of CK) using a protocol described previously with some minor modification (Tarkowski et al., 2009). 10 μl extracts were injected for analysis using an LC–MS/MS (Quantum Access, Thermo Scientific, USA). The leaves (0.1–0.2 g) of *AtCKX3* inducible lines, *AtNAP* inducible lines and WT 0 and 4 days after DEX induction were used. The DEX induction was performed as previously described (Guo and Gan, 2006; Zhang et al., 2012).

### Supplementary Information

**Additional file 1.** Table S1 Primers used in the research.

**Additional file 2. **Supplemental Fig. S1 qPCR analyses of *AtCKX1, 2, 4, 5, 6* during leaf senescence in Arabidopsis.

**Additional file 3. **Supplemental Fig. S2 OsNAP (rice) and AtNAP could bind to the promoter of *OsCKX8* but not *OsCKX9* as revealed by yeast one-hybrid analyses.

## Data Availability

The materials are available.

## Abbreviations

ABA: abscisic acid
At: *A. thaliana*
CKs: cytokinins
CKX: cytokinin oxidase/dehydrogenase
DAG: days after germination
DEX: dexamethasone
GAD: GAL4 activation domain
HAN: HANABA TARANU
IPA: isopentenyladenosine
LC–MS/MS: liquid chromatography with tandem mass spectrometry
NAP: NAC-LIKE, ACTIVATED BY AP3/P1
PP2C: protein phosphatase 2C
SAGs: senescence-associated genes
TF: transcription factor
