# Supplementary material for: The leaf senescence-promoting transcription factor AtNAP activates its direct target gene CYTOKININ OXIDASE 3 to facilitate senescence processes by degrading cytokinins
Source: Mol Hortic. 2021 Oct 13;1:12. doi: 10.1186/s43897-021-00017-6 (PMC10515059; doi:10.1186/s43897-021-00017-6)
Supplement: Supplementary file 1 — Additional file 1. Table S1 Primers used in the research. [file 43897_2021_17_MOESM1_ESM.pdf]

Table S1 Primers used in this research

Primer names	Nucleotide sequences (from 5'to 3')	Purposes
G4010	CTCGAGTTTTTTTGAGAAATAAGTCTTTTT	Forword primer to clone AtCKX3 promoter for Y1H
G4011	GAATTCGTCAAGTTTAACTCTCACAAAA	Reverse primer to clone AtCKX3 promoter for Y1H
G4036	GAATTCTTTATGCAGAACCATTTAACTAGTG	Reverse primer to clone preceding part of AtCKX3 promoter without binding motif for Y1H
G4037	CTCGAGTTTCAATAGACCACAAAAGC	Forword primer to clone latter part of AtCKX3 promoter with binding motif for Y1H
G4038	CTCGAGTCACCTCCAACTAAGATCTAAT	Forword primer to clone AtCKX1 promoter for Y1H
G4039	GAATTCTTCTACTTTGTTGAGAGAAATTGC	Reverse primer to clone AtCKX1 promoter for Y1H
G4387	TCTACATGTCAGTTGATCATTCATTGTAAA	Reverse primer to clone first part of AtCKX3 promoter with transversion mutations
G4388	CAACTGACATGTAGAATCTAATTAGCAAACC	Forword primer to clone second part of AtCKX3 promoter with transversion mutations
G4389	TCTTTGATCATTCATTGTAAA	Reverse primer to clone first part of AtCKX3 promoter with deletion mutations
G4390	CAAAGAATCTAATTAGCAAACC	Forword primer to clone second part of AtCKX3 promoter with deletion mutations
G3947	CCATGGTTTTTTTGAGAAATAAGTC	Forword primer to clone AtCKX3 promoter for GUS assay
G3948	CTGCAGGTTTGAATATTCCATAGAA	Reverse primer to clone AtCKX3 promoter for GUS assay
G4042	GGTACCCTAACTCGAGTTTATTTTTTGA	Forword primer to clone AtCKX3 CDS for gene complementary
G4043	CTGCAGATGGCGAGTTATAATCTTC	Reverse primer to clone AtCKX3 CDS for gene complementary
G4044	CTGCAGTTTTTTTGAGAAATAAGTCTTTT	Forword primer to clone AtCKX3 promoter for gene complementary
G4045	AAGCTTAGATCGTAAAATATATTCAACAGGTA	Reverse primer to clone AtCKX3 promoter for gene complementary
G3945	ACTAGTATGGCGAGTTATAATCTTCGTTC	Forword primer to clone AtCKX3 CDS for DEX induced expression
G3946	CTGCAGCTAACTCGAGTTTATTTTTTG	Reverse primer to clone AtCKX3 CDS for DEX induced expression
G3897	TTTAATGCGTCTACGTTTACAACC	Forword primer to identify *atcks3* knockout mutation
G3898	ACTCTTCATTGATAAGAATCAAGCT	Reverse primer to identify *atcks3* knockout mutation
G3899	TATGAATCGCAACAAGTGGAATG	Forword primer to test transcription of *AtCKX3* for qPCR
G3900	CCTCGGACCAAAATGTCTAACC	Reverse primer to test transcription of *AtCKX3* for qPCR
Actin2 F	AGTGGTCGTACAACCGGTATTGT	Forword primer to test transcription of *Actin2* for qPCR
Actin2 R	GATGGCATGAGGAAGAGAGAAAC	Reverse primer to test transcription of *Actin2* for qPCR
G3149	CGTAAAGCATCAACGAAACG	Forword primer to test transcription of *AtNAP* for qPCR
G3835	TGGAAGTTTCATCGACGTCAT	Reverse primer to test transcription of *AtNAP* for qPCR
G4150	TTCTTGGCGGGCTTGGAC	Forword primer to test transcription of *AtCKX1* for qPCR
G4151	GACTTGAATCTGCTTGCCTGTG	Reverse primer to test transcription of *AtCKX1* for qPCR
G4152	CTTGTCTTTTGAAACCACCACG	Forword primer to test transcription of *AtCKX2* for qPCR
G4153	CATCATCTCCGCAGCCTCT	Reverse primer to test transcription of *AtCKX2* for qPCR
G4154	CCGTCCTCTGCCCTTCCT	Forword primer to test transcription of *AtCKX4* for qPCR
G4155	CCCGTCTGCCGAGATAACAA	Reverse primer to test transcription of *AtCKX4* for qPCR
G4156	TCTCCACCCCATACATCCACA	Forword primer to test transcription of *AtCKX5* for qPCR
G4157	CCCTTCCGACTTAGCCTCC	Reverse primer to test transcription of *AtCKX5* for qPCR
G4158	GCCCAGTCATCGTCTACCCA	Forword primer to test transcription of *AtCKX6* for qPCR
G4159	CCCCACTTGTCCCCGAAA	Reverse primer to test transcription of *AtCKX6* for qPCR
