# Supplementary material for: The leaf senescence-promoting transcription factor AtNAP activates its direct target gene CYTOKININ OXIDASE 3 to facilitate senescence processes by degrading cytokinins
Source: Mol Hortic. 2021 Oct 13;1:12. doi: 10.1186/s43897-021-00017-6 (PMC10515059; doi:10.1186/s43897-021-00017-6)
Supplement: Supplementary file 2 — Additional file 2. Supplemental Fig. S1 qPCR analyses of AtCKX1, 2, 4, 5, 6 during leaf senescence in Arabidopsis. [file 43897_2021_17_MOESM2_ESM.pdf]

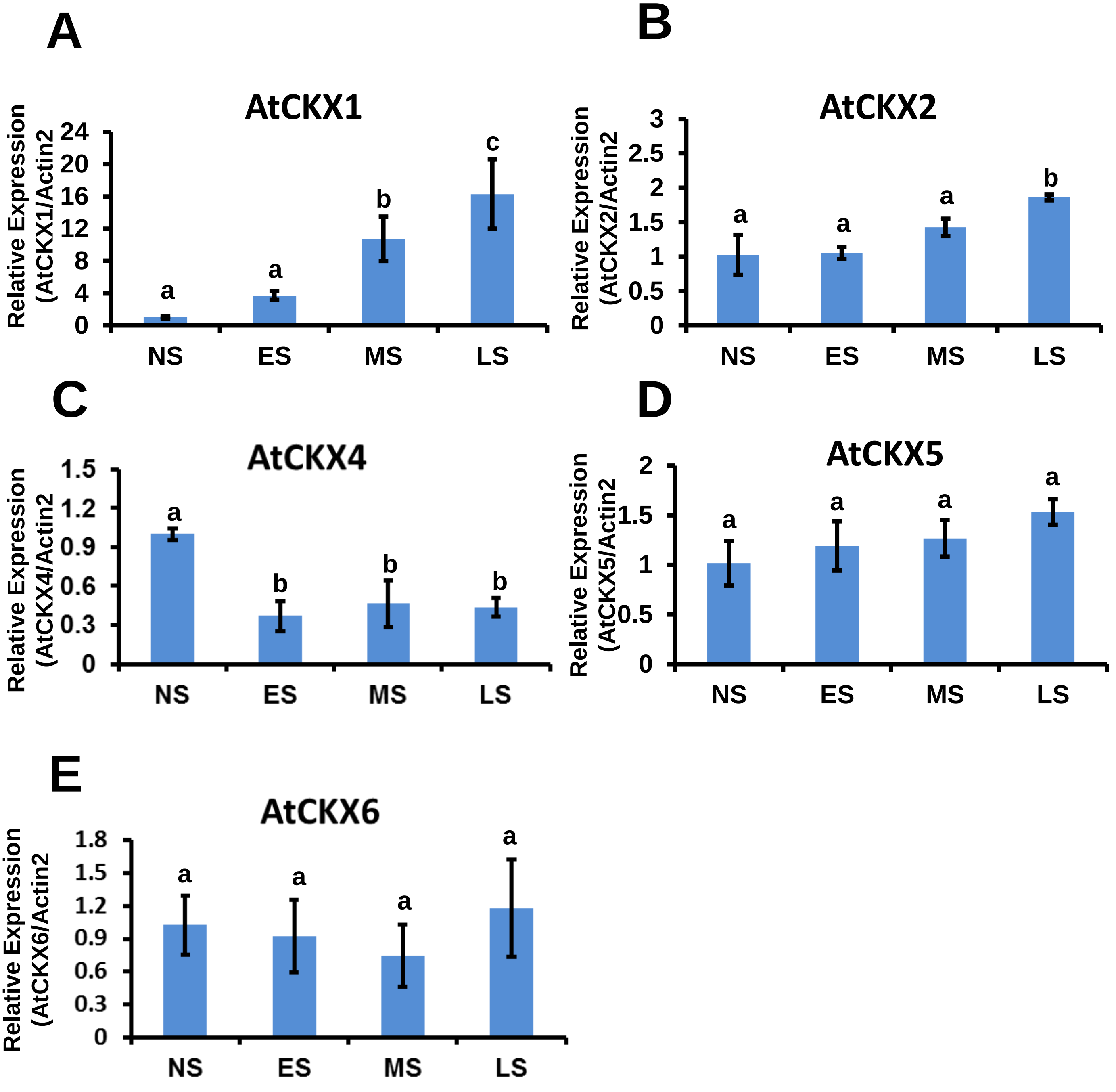

Fig. S1 Transcription of cytokinin oxidase/dehydrogenase genes in *Arabidopsis* during leaf senescence. (A)-(E)) qPCR analysis testing the relative expression of *AtCKX1*(A), *AtCKX2*(B), *AtCKX4*(C), *AtCKX5*(D) and *AtCKX6*(F) during leaf senescence in wild type plant. NS, leaves at fully expanded stage; ES, leaves at early senescence stage, less or equal 25% yellowing; MS, leaves at middle senescence stage, about 50% yellowing; LS, leaves at late senescence stage, greater or equal 75% yellowing. Data indicate mean values ± SD of three samples. Significant (P<0.05) differences between means are indicated by different letters using Tukey's HSD test.
