# Supplementary material for: The leaf senescence-promoting transcription factor AtNAP activates its direct target gene CYTOKININ OXIDASE 3 to facilitate senescence processes by degrading cytokinins
Source: Mol Hortic. 2021 Oct 13;1:12. doi: 10.1186/s43897-021-00017-6 (PMC10515059; doi:10.1186/s43897-021-00017-6)
Supplement: Supplementary file 3 — Additional file 3. Supplemental Fig. S2 OsNAP (rice) and AtNAP could bind to the promoter of OsCKX8 but not OsCKX9 as revealed by yeast one-hybrid analyses. [file 43897_2021_17_MOESM3_ESM.pdf]

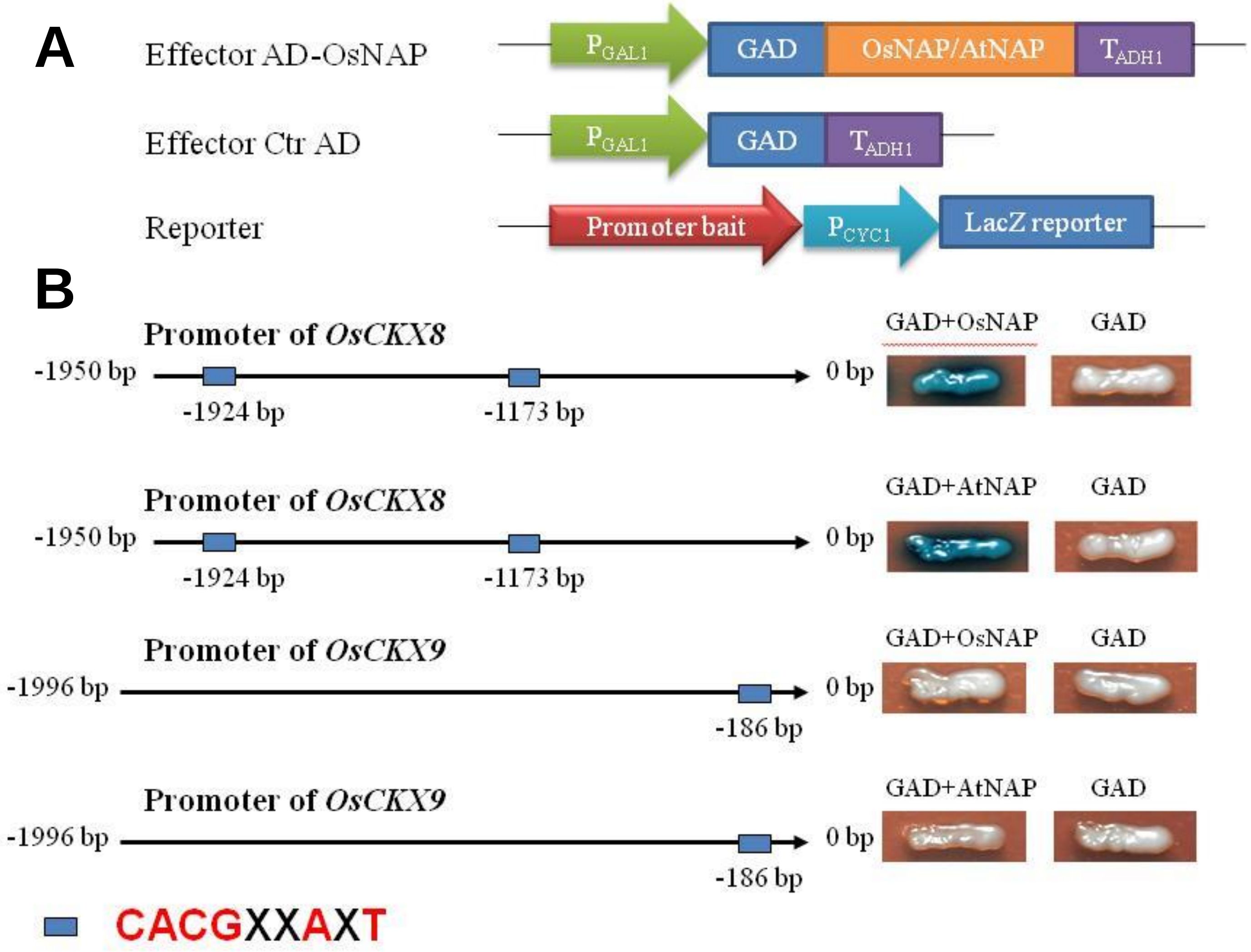

Fig. S2 Physical interaction of OsNAP with the OsCKX8 promoter revealed by yeast one-hybrid assay. (A) Schematic representation of the constructs in the studies. The expression of prey GAD fused with or without (negative control or Ctr) AtNAP or OsNAP were driven by the GAL1 promoter (pGAL1). The LacZ gene driven by OsCKX8 or OsCKX9 promoter served as the bait. (B) Activation of the LacZ reporter by binding of the fusion protein GAD-AtNAP or GAD-OsNAP to the OsCKX8 promoter. Blue color suggests a binding, white color suggests no binding. The sequence in red represents the motif which AtNAP binds to. A of the translation start site was numbered as +1.
